# Diminished acquired equivalence yet good discrimination performance in older participants

**DOI:** 10.3389/fpsyg.2013.00726

**Published:** 2013-10-11

**Authors:** Jasper Robinson, Emma Owens

**Affiliations:** School of Psychology, The University of Nottingham, Nottingham, UK

**Keywords:** acquired equivalence, attentional set, ageing, discrimination learning, connectionism, associative learning, healthy aging, configural processing

## Abstract

We asked younger and older human participants to perform computer-based configural discriminations that were designed to detect acquired equivalence. Both groups solved the discriminations but only the younger participants demonstrated acquired equivalence. The discriminations involved learning the preferences [“like” (+) or “dislike” (−)] for sports [e.g., tennis (t) and hockey (h)] of four fictitious people [e.g., Alice (A), Beth (B), Charlotte (C), and Dorothy (D)]. In one experiment, the discrimination had the form: At+, Bt−, Ct+, Dt−, Ah−, Bh+, Ch−, Dh+. Notice that, e.g., Alice and Charlotte are “equivalent” in liking tennis but disliking hockey. Acquired equivalence was assessed in ancillary components of the discrimination (e.g., by looking at the subsequent rate of “whole” versus “partial” reversal learning). Acquired equivalence is anticipated by a network whose hidden units are shared when inputs (e.g., A and C) signal the same outcome (e.g., +) when accompanied by the same input (t). One interpretation of these results is that there are age-related differences in the mechanisms of configural acquired equivalence.

## Introduction

Experiments on “*acquired equivalance*” have revealed important information about the way in which animals encode stimulus representations. For example, Honey and Ward-Robinson (2001) gave rats acquired equivalence training in which a tone would signal food delivery (t+) and a clicker would not (c−) in two distinctly decorated Skinner boxes (A and C). But in two other Skinner boxes (B and D), the tone and click signalled the alternative outcome (i.e., t− and c+). The complete discrimination can be represented as: At+, Ac−, Bt−, Bc+, Ct+, Cc−, Dt−, Dc+. It was evident that rats had learned the discrimination because they anticipated the delivery of food on reinforced (+) trials by approaching the site of delivery and refrained from this on the non-reinforced (−) trials. Notice that no single stimulus uniquely predicts either outcome: all stimuli are equally often reinforced and non-reinforced and it is necessary for rats to learn about specific *configurations* of stimuli. Influential theoretical accounts of such learning (e.g., Rescorla, 1976; Pearce, 2002) provide accounts of solution of the At+, Ac−, Bt−, Bc+, Ct+, Cc−, Dt−, Dc+ discrimination in which the eight trial types are represented by eight “configural” stimuli, each being associated with the appropriate outcome. However, Honey and Ward-Robinson gave an additional stage of training that produced results not anticipated by these models. In the subsequent stage, rats were split into two groups to receive different types of “reversal training,” in which at least some of the trial outcomes were switched. For group Whole, all trial types were reversed (i.e., At−, Ac+, Bt+, Bc−, Ct−, Cc+, Dt+, Dc−) but for group Part only half of the trial types were reversed (At+, Ac−, Bt−, Bc+, Ct+, Cc−, Dt+, Dc−). Both groups' performances were reduced by the reversal from the original stages and both recovered; however, group Whole's performance recovered more quickly than group Part's did. It is this feature of the data that challenges alternative configural learning theories (e.g., Rescorla, 1976; Pearce, 2002). Notice that in the pre-reversed discrimination these Skinner boxes indicate the equivalent reinforcement arrangements for the tone and click. Informally expressed, it is as though rats' representations of Skinner boxes A and C (and B and D) had “acquired equivalence” during pre-reversal training. Thus, new learning during the reversal may transfer between A and C (and between B and D). For group Part, the acquired equivalence between A and C (and between B and D) will lead to conflicting information because A and C no longer indicated equivalent tone/click reinforcement relationships. But for group Whole, although the tone/click reinforcement relationships have all reversed, A and C (and B and D) remain equivalent. It is notable that non-configural forms of acquired equivalence are possible (e.g., Honey and Hall, 1989) but they are interpretable in simpler terms than those considered here (e.g., Ward-Robinson and Hall, 1999).

Honey et al. (2010) describe this finding, and others like them (e.g., Ward-Robinson and Honey, 2000; Hodder et al., 2003), in terms of a three-layer connectionist network, which will be described in detail in the Discussion. Those authors also note that their model will adequately explain the finding that discriminations involving “intra-dimensional” shifts are mastered more quickly than those involving “extra-dimensional” shifts (e.g., Owen et al., 1991; Barense et al., 2002). This suggestion is theoretically significant because, if substantiated, it would undermine claims that non-human animals' demonstrations of intra-dimensional transfer are not actually demonstrations of a genuine attentional process (cf., Mackintosh, 1974). It is also clinically significant because intra-/extra-dimensional shift experiments in rats demonstrate the role of the prefrontal cortex in “attentional set” in frontal lobe disorders (Owen et al., 1991; Dias et al., 1997; Birrel and Brown, 2000; Hampshire and Owen, 2010). Deficits in performance in intra-/extra-dimensional shift have been reported in apparently healthy, older human volunteers (Owen et al., 1991; see also, Barense et al., 2002). That observation and Honey et al.'s assertion that the same psychological processes outlined in their model, govern not only acquired equivalence and intra-/extra-dimensional set shifting, make several predictions. In particular, manipulations that affect intra-/extra-dimensional set shifting, should also affect acquired equivalence. We report here results of two experiments that support that prediction by demonstrating acquired equivalence performance to be diminished in (healthy) older participants relative to younger participants.

## Experiment 1

**Figure 1:**
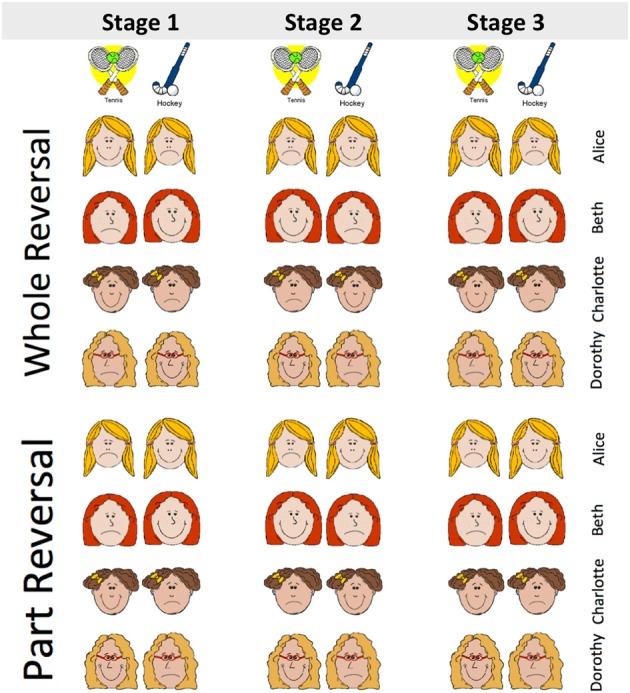
**Example of treatments given to the younger and older participants in Experiment 1.** Participants are required to learn whether four fictitious characters, Alice, Beth, Charlotte, and Dorothy, like or dislike the sports tennis and hockey. In each of three stages, two characters like and two dislike each of the two sports; these patterns of liking and disliking are complemented by the remaining two characters. In the example of a “whole reversal” treatment in the top panel, the stage-2 treatment consists of a full reversal of the pattern of the characters' liked and disliked sports. But in the example of a “part reversal” only two of the characters' liked and disliked two sports reverses (viz., Beth, and Charlotte), whereas the other two characters' (viz., Alice, and Dorothy) liked and disliked sports remain unchanged from stage 1. Stage-3 training was identical to Stage-1 training, and was intended to offer an additional attempt to examine the effect of whole or part reversal.

Honey and Ward-Robinson (2001) demonstrated acquired equivalence in rats using an appetitive conditioning procedure. We adapted their procedure for use with older and younger participants in Experiment 1 whose design is summarized in Figure 1. Older and younger participants were required to learn about four fictitious characters' like or dislike of two sports. Two of the characters liked the same two sports and disliked the two alternative sports (Stage 1). Acquired equivalence could be demonstrated over a series of “reversals” (Stage 2 and Stage 3) in which some or all of the previously liked sports became disliked and vice versa. If the two pairs of characters had acquired equivalence, participants' performance should recover more rapidly from the whole reversal than from the part reversal. The new question we asked here was: would this acquired equivalence effect be different in a group of older participants?

## Materials and methods

### Participants

Group Y comprised two men and fourteen women with a mean age of 20.8 years (range: 20–24 years); Group O comprised seven men and eight women with a mean age of 64.4 years (range: 55–77 years). Participants were a self-selected sample of respondents to recruitment posters in public places (cafés, Post Offices, etc., Group O) or were University of Nottingham students who gained course credit for participation (Group Y). All participants were naive with respect to the stimuli used in the experiment.

### Apparatus and stimuli

Experiments were run in a small quiet room in the School of Psychology, University of Nottingham. Stimuli were presented and responses were recorded on a laptop (Toshiba Portégé A200). From opposite corners its screen measured 31 cm. Participants used a separate keyboard that was connected to the laptop and positioned such that the participant could use the keyboard while looking at the laptop screen. The keyboard consisted of a standard QWERTY keyboard with a number pad to the right hand side. With the exception of the keys numbered 1, 2, 3, 4, and 5 that ran along the top of the QWERTY part of the keyboard and the space key, black stickers covered the letter/number of each key. Our intention here was to direct participants' responses to the keys 1, 2, 3, 4, and 5 during the experiment. A pair of headphones (Panasonic RP-HT225) was plugged into the laptop and was used to present the auditory stimuli described below.

The following cartoon depictions were used as stimuli: (a) a pair of crossed tennis rackets and ball with “Tennis” written below them; (b) a hockey stick and ball with “Hockey” written below them; (c) four characters' faces with “neutral” facial expressions, “happy” facial expressions and “sad” facial expressions (i.e., twelve images of the characters). Each character's neutral image had her name (Alice, Beth, Charlotte or Dorothy) written below it. Each character's happy image had “X Likes this Sport” written above it where X is the character's name; sad images were similarly accompanied by text that read “X Dislikes this Sport.” These stimuli occupied a screen area of around 400 mm^2^. Auditory data files had been created on a computer (iMac, Apple Computers) using a synthetic voice (“Victoria”). These files read aloud the text “Correct,” “Incorrect” and “Have a guess next time” and had durations of between 0.5 and 1.5 s. During the experiment, the character and sport stimuli were presented side by side and vertically central. The character appeared only on the left-hand side; the sport appeared only on the right-hand side. A numbered scale comprising the numbers 1, 2, 3, 4, and 5 could be presented below the neutral images of the characters' faces with 1 on the left and 5 on the right. The word “Dislike” appeared to the left of “1” and the word “Like” appeared to the right of “5.”

### Procedure

Participants from Groups Y and O were randomly assigned to whole-reversal (W) or part-reversal (P) groups, to create Group YW, Group YP, Group OW, and Group OP, see Figure 1. Participants' mean ages in groups YW, YP, OW, and OP were, respectively 20.1, 21.5, 64.6, and 64.3 years. There were seven woman and one man in both Group YW and YO; there were five women and two men in Group OW; and there were three women and five men in Group OP.

All participants were given training in which they were asked to learn which sports (Tennis and Hockey) four fictitious characters (Alice, Beth, Charlotte, and Dorothy) liked. Participants keyed “5” for liked sports and “1” for disliked sports. Keys in-between could be used for less confident responses. For the purposes of feedback (see below), keying 4 or 5 were “correct” on like trials and “incorrect” on dislike trials; and keying 1 or 2 were “correct” on dislike trials and “incorrect” on like trials. Keying 3 was neither correct nor incorrect. For all participants, each sport was liked by two of the characters and disliked by the other two characters; each character liked one sport and disliked the other. Thus, each character agreed in her opinion of the two sports with one other character and disagreed with the two remaining characters. We counterbalanced stimulus arrangements such that for some participants Alice and Charlotte (and therefore, Beth and Dorothy) had equivalent sports opinions and for others Alice and Beth (and therefore, Charlotte and Dorothy) has equivalent sports opinions. Neither Alice and Dorothy nor Beth and Charlotte shared sports opinions for any participants. For some participants the shared opinions were based on liking Tennis (and, therefore, disliking Hockey); for others participants the shared opinions were based on liking Hockey (and therefore, disliking Tennis). The orthogonal arrangement of this counterbalancing created four different discriminations, which were given to similar numbers of participants.

Participants read a standard instruction sheet that gave an indication of the rationale of the experiment and emphasized participants' entitlement to leave the experiment. Instructions were then presented on the laptop. A scenario was described involving the participant learning which of two sports the four characters liked. Instructions described making 1–5 key responses to indicate each character's like/dislike of the sports. At the end of the instruction phase the experimenter went through an example of how to use the keyboard to register responses. The experimenter checked that the participant understood and was comfortable with the task, and then left the room. The instruction phase repeated then the participants pressed the spacebar to initiate the trials.

**Figure 2:**
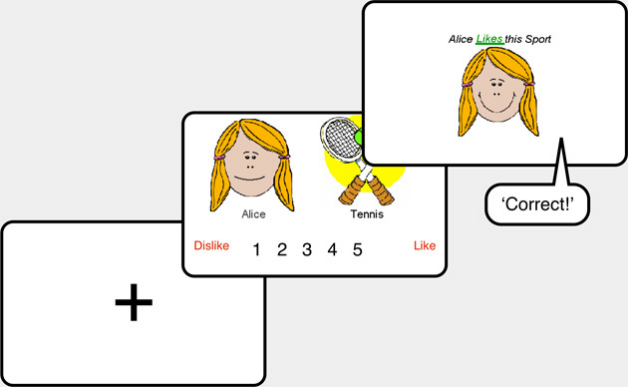
**Illustration of the the sequence of events on each trial of training in Experiments 1 and 2.** (1) The leftmost panel represents the presentation on the computer's screen of the fixation cross (“+”), which occurred at the beginning of each trial for 1.5 s. There was no requirement of the participant; (2) The central panel represents the presentation on the computer screen of the character, the sport and the rating scale. This example represents a trial in which a participant was asked to rate Alice's like/dislike of tennis, however, other trial types occurred (see, e.g., Figures 1, 3). This slide remained until the participant had made their rating; (3) The rightmost panel represents the feedback given to the participant following their previous rating. In this example, the participant correctly rated Alice as liking tennis (i.e., the participant gave Alice a rating of ≥4 for tennis), which was accompanied by the spoken word “Correct” and by the statement “Alice likes this sport.” Full details of the feedback given on incorrect trials and on the other types of trial are given above.

The sequence of one type of trial is exemplified in Figure 2. Each trial consisted of: (1) The 1.5-s, centrally located presentation of the keyboard character “+,” (2) The presentation of a character and a sport, during which the participant had unlimited time to select a response from 1 (dislike) to 5 (like) of the scale that was presented below them, (3) Information about the characters like/dislike of the sport was given for 2.9 s. This comprised presentation of text (e.g., Alice likes this Sport) with the accompanying “happy” or “sad” version of the character and the Tennis or Hockey picture, (4) Auditory feedback was given (“Correct,” “Incorrect” or, where 3 was keyed, “Have a guess next time”).

During stage-1 training, each of the eight trial types was given 24 times in an irregular sequence (i.e., 96 liked and 96 disliked trials). During stage-2 training, participants received an identical treatment but on a reversed version of the task: for participants in Groups YW and OW, all four of the characters now liked the sports they had previously disliked and now disliked the sports they had previously liked. For participants in Groups YP and OP, only two of the characters' opinions of the sports reversed. Stage 3 was the final stage of training and was identical to the first except that only sixteen trials of each of the eight trial types was given. Group YP and OPs' partial reversals were systematically varied and were arranged so that each particular discrimination was matched by a subgroup of YW and OW. Thus, performance differences between whole/part reversals could not be attributed to differences in difficulty of the specific trials types in their discrimination.

## Results

**Figure 3:**
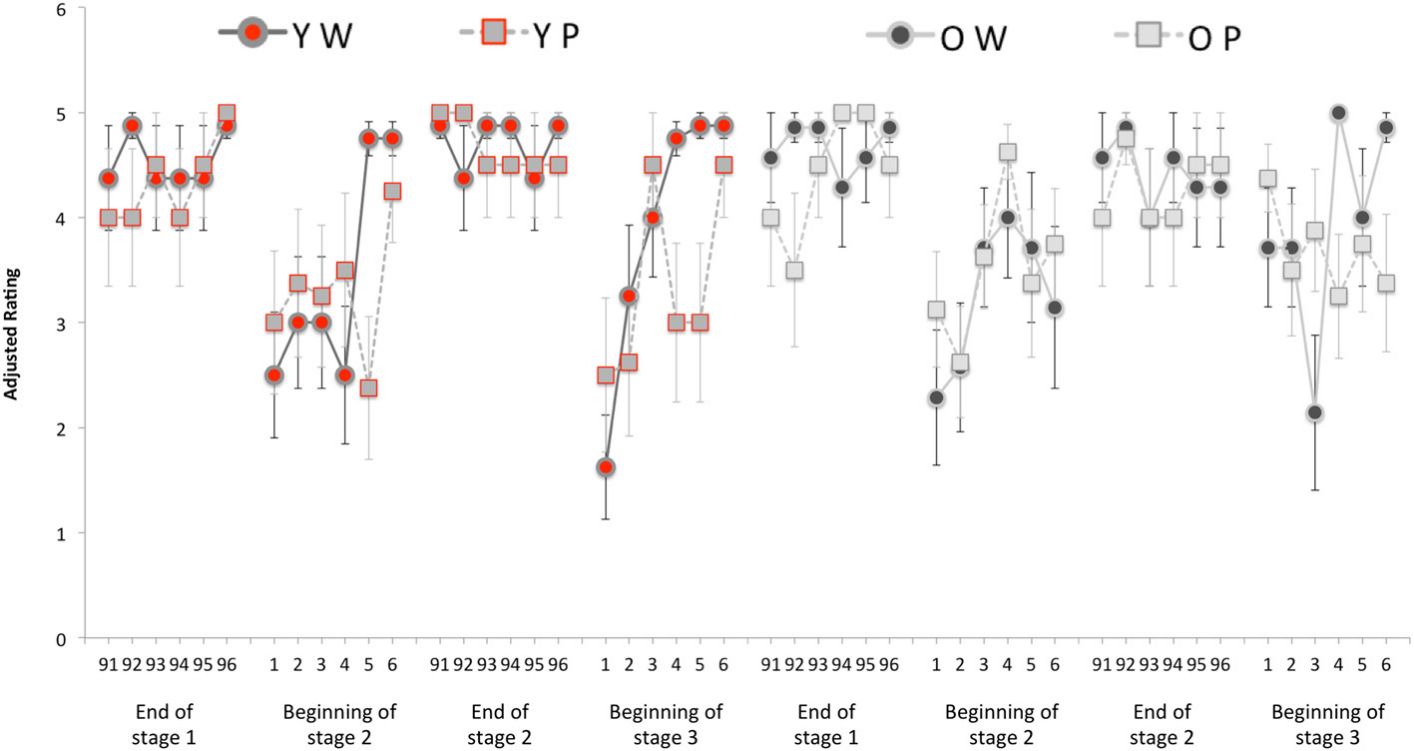
**Means, and one standard error of each mean, of data from Experiment 1.** The leftmost four sets of six trials of data are from the two younger participant groups (groups YW and YP); the rightmost data are the corresponding data from two older participant groups (groups OW and OP). For all four groups, and running from left to right, the four sets of data represent: (1) The final six liked and final six disliked trials of stage 1 (i.e., trials 91 through to 96 of stage 1); (2) the first six liked and first six disliked trials of stage 2 (i.e., trials 1 through to 6 of stage 2); (3) the final six liked and final six disliked trials of stage 2 (i.e., trials 91 through to 96 of stage 2); (4) the first six liked and first six disliked trials of stage 3 (i.e., trials 1 through to 6 of stage 3). The ratings are expressed in a like/dislike-independent form such that data from dislike trials were transformed to match the scale of the like trials. Thus, here data are pooled over the like and dislike trial types and scores of 5 is (maximally) correct and a score of 1 is (maximally) incorrect.

Initial examination of raw trial-by-trial data revealed that all participants mastered the task rapidly, for example reaching asymptotic discrimination after about six blocks of the eight trial types. Our analysis focuses, therefore, on the terminal six trials of established training and the initial six trials of the reversal. Here, group differences were not masked by rapid discrimination learning. Data on “dislike” trials were transformed to match the scale of the “like” trials. That is, 1 (the correct response) was recoded as 5, 2 as 4, 3 remained as 3, 4 as 2 and 5 as 1. This obtained a like/dislike-independent response measure in which 5 s indicate the correct response and 1 s indicate the incorrect response. These data are summarized in Figure 3. We see that all four groups' performance before both reversals was good (around the asymptote of 5) and that it declined on both of the reversals, recovering quickly. Inspection of the two Y groups, indicates that group YW recovered from the disruption of the reversal more quickly than group YP. However, no such pattern can be seen in the O groups: groups OW and OP show no obvious difference in recovery. This description of the data was supported by an analysis of variance (ANOVA) with within-subject variables of: (1) cycle (i.e., the first and second twelve-trial cycles of established discrimination and subsequent reversal), (2) established training (end of stages 1 and 2) versus reversal stage (beginnings of stages 2 and 3), and (3) trial; and between-subject variables of: (1) age (i.e., Y versus O), and (2) reversal group (i.e., W versus P). The analysis revealed main effects of trial, *F*_(5, 135)_ = 4.9, *p* < 0.001, η^2^_p_ = 0.156, reversal stage, *F*_(1, 27)_ = 40.7, *p* < 0.001, η^2^_p_ = 0.602 and cycle, *F*_(1, 27)_ = 5.0, *p* < 0.034, η^2^_p_ = 0.158. The Cycle × Trial × Age interaction, *F*_(5, 135)_ = 2.9, *p* < 0.017. η^2^_p_ = 0.097, and the Cycle × Trial × Reversal Stage interaction, *F*_(5, 135)_ = 2.3, *p* < 0.046, η^2^_p_ = 0.079 were significant. No other main effect was significant. The source of the interaction involving the age variable was examined using similar analyses separated for young and older participants. Analysis of older participants' data yielded a main effect of reversal stage only, *F*_(1, 13)_ = 15.0, *p* < 0.003, η^2^_p_ = 0.537. No other statistic was significant and, of most importance, none was significant that involved the reversal-group variable, smallest *p* > 0.121. However, the corresponding analysis of the younger participants' data yielded reliable main effects of cycle, reversal, and trial, and reliable Reversal × Trial, and Reversal × Trial × Reversal Group interactions, largest *p* < 0.024, *F*_(5, 70)_ = 2.7, η^2^_p_ = 0.167. The source of the younger participants' Reversal × Trial × Reversal Group interaction was examined using a pair of ANOVAs with data split across the reversal stage variable (i.e., on established discrimination data and reversed data) with only cycle and reversal group as variable. No significant statistics were obtained for the established discrimination data, smallest *p* > 0.134. The corresponding ANOVA for the reversed data yielded a significant main effects of cycle and trial and a significant Trial × Reversal Group interaction, largest *p* < 0.012, *F*_(1, 29)_ = 7.3, η^2^_p_ = 0.203. Simple main-effects analysis on this interaction using separate error terms for each trial, revealed younger participants' whole reversal performance to be superior to partial reversal performance on the fifth trial, *F*_(1, 14)_ = 13.7, *p* < 0.003, η^2^_p_ = 0.495.

## Experiment 2

**Figure 4:**
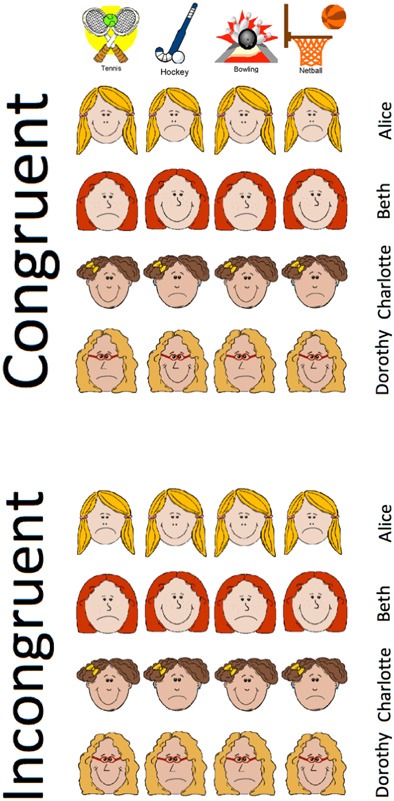
**Example of treatments given to the younger and older participants in Experiment 2.** Participants are required to learn whether four fictitious characters, Alice, Beth, Charlotte and Dorothy, like or dislike the sports tennis, hockey bowling, and netball. Unlike Experiment 1, training consisted of the single stage represented here. Two characters like two of the four sports and dislike the other two sports; these patterns of liking and disliking are complemented by the remaining two characters. In the example of a “congruent” treatment in the top panel, two pairs of characters like the same two sports (Alice and Charlotte both like tennis and bowling, and Beth and Dorothy both like hockey and netball) and dislike the same sports (Alice and Charlotte both dislike hockey and netball, and Beth and Dorothy both dislike tennis and bowling).But in the example of an “incongruent” treatment in the bottom panel, no two pairs of characters share patterns of liking and disliking of the sports. For example, although Alice and Beth both like hockey and dislike tennis, Alice likes bowling, whereas Beth dislikes it.

The results of Experiment 1 join those of Honey and Ward-Robinson (2001) and Hodder et al. (2003) in showing an acquired equivalence effect by an improved rate of “whole” reversal learning relative to “part” reversal learning in younger participants. Our new finding is that this difference in whole/part learning rate was absent in older participants. Before considering fully the implications of this finding, we sought to replicate it using similar logic to that of Experiment 1. For Experiment 1 to reveal acquired equivalence, it is necessary for the benefit of acquired equivalence to more than offset the cost of relearning new character-sport relationships. In Experiment 2, which is summarized in Figure 4, we followed Honey and Ward-Robinson (2001) in the use of a design that avoids this compromise. Older and younger participants were required to learn the four characters like/dislike of four sports. For the Congruent treatment, each of the characters' like/dislike of the the four sports was matched with one other character. For the Incongruent treatment, no one character's sport like/dislike was matched with any other character. Acquired equivalence could be demonstrated by the finding that the discrimination was mastered more rapidly in the congruent than the incongruent condition. Again, we asked whether the extent of acquired equivalence would be different in the two age groups.

## Materials and methods

### Participants, apparatus, and stimuli

Group Y comprised six men and ten women with a mean age of 21.2 years (range: 18–24 years); Group O comprised five men and eleven women with a mean age of 64.8 years (range: 53–77 years). The apparatus and stimuli were those used in Experiment 1. Experiment 2 used an additional two sport stimuli, bowling and netball to make a total of four sports for the four characters. All unspecified details of participants, apparatus and stimuli were identical to those of Experiment 1.

### Procedure

Participants from Groups Y and O were randomly assigned to congruent (C) or incongruent (I) groups, to create Group YC, Group YI, Group OC, and Group OI. The mean ages and numbers of women and men in these groups was, respectively: 21.0, 22.5, 64.5, and 65.1 years; and 6:2, 4:4, 5:3, and 6:2. All participants were given training in which they were asked to learn which of the four sports (Tennis, Hockey, Bowling, and Netball) the four fictitious characters (Alice, Beth, Charlotte, and Dorothy) liked. For all participants, each of the four sports was liked by two of the characters and disliked by the other two characters; each character liked two sports and disliked the other two sports. For the congruent groups, each character shared her pattern of sport liking and disliking with one other character and the two remaining characters had the complementary pattern of liking and disliking of sports. For all participants in the congruent groups Alice was equivalent to Charlotte and Beth was equivalent to Dorothy. For approximately half of the participants in the two congruent groups this was based upon Alice and Charlotte's shared liking of Bowling and Tennis (and shared disliking of Netball and Hockey); for the remainder of the participants in the two congruent groups, equivalence was based upon Alice and Charlotte's shared disliking of Bowling and Tennis (and their shared liking of Netball and Hockey). The arrangements of Alice and Charlotte's liking and disliking of Bowling and Netball was the same for the two incongruent groups as for the two congruent groups. The incongruent groups' treatment differed from the congruent groups' treatment in the four characters' liking and disliking of Tennis and Hockey: for approximately half of the participants in the two incongruent groups, Alice and Beth liked Tennis (and disliked Hockey); but for the remainder Alice and Beth disliked Tennis (and liked Hockey). Notice that for the incongruent groups no two characters were exactly alike in their pattern of sports liking.

All participants received 256 trials in random sequence with the constraint that each of the sixteen trial types created by the combinations of the four characters and four sports occurred once in each block of sixteen trials. Unspecified procedural details were identical to those of Experiment 1.

## Results

**Figure 5:**
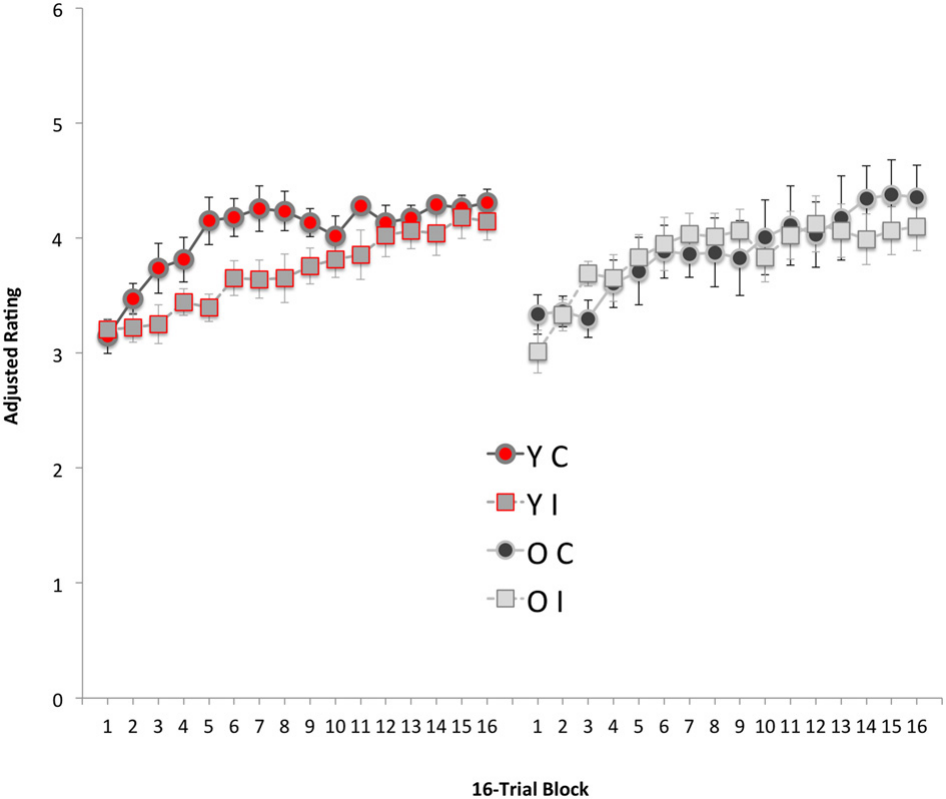
**Means, and one standard error of each mean, of data from Experiment 2.** The leftmost 16 8-trial blocks of data are from the two younger participant groups (groups YC and YI); the rightmost data are the corresponding data from two older participant groups (groups OC and OI). The ratings are expressed in a like/dislike-independent form such that data from dislike trials were transformed to match the scale of the like trials. Thus, here data are pooled over the like and dislike trial types and scores of 5 is (maximally) correct and a score of 1 is (maximally) incorrect.

The results of Experiment 2 are summarized in Figure 5. As in Experiment 1, the scale for dislike trials was reversed to match that of the like trials and data were collapsed over like and dislike trials. Initial inspection and analysis revealed that the older participants' discrimination performance was robust, though the response buttons were often not the most extreme (i.e., responses of 1 and 5). This feature of the data indicates that older participants may have differed from younger participants in their response bias (i.e., tending to make more accurate, but more modest, responses). For example, on the 16th block of training, only three of the sixteen younger participants gave mean responses that were not 5 s or 1 s, however, at that point, fifteen of the sixteen older participants gave scores that were not 5 s or 1 s (χ^2^ = 15.4, *p* < 0.001). To correct for this bias each datum was normalized by multiplying it by a normalization ratio (cf., Ringo, 1988; Baxter and Murray, 2001). The normalization ratio was computed for each block by dividing the arithmetic mean of all data for that block (i.e., ignoring age and congruency designation) by the mean for the age group (i.e. ignoring only congruency designation) on that block. This process acted to moderate younger participants' responses and boost older participants' responses, which was irrespective of congruency designation.

We see that participants in all groups learned the relationships between the characters and the sports and, it seems, less rapidly than the discrimination in Experiment 1, which could be the result of the additional number of trial types. Group YC appeared to master the discrimination more rapidly than Group YI. The question of key interest is whether the older participants would also show acquired equivalence. In particular, would Group OC's performance show superiority over Group OI's? As in Experiment 1, the older participants appear to have satisfactorily learned the discrimination but do not demonstrate acquired equivalence. This description of the data was supported by an ANOVA with block as a within-subject variable and age and congruency as between-subject variables, which revealed a main effect of block, *F*_(15, 420)_ = 16.6, *p* < 0.001, η^2^_p_ = 0.373 and a Block × Age × Congruency interaction, *F*_(15, 420)_ = 1.8, *p* < 0.035, η^2^_p_ = 0.060. No other statistics were significant, smallest *p* > 0.216, *F*_(1, 28)_ = 1.6, η^2^_p_ = 0.054.

The source of the Block × Age × Congruency interaction was located by performing a pair of separate, 2 × 16 ANOVAs on younger and older participants' data. The ANOVA on the younger participants' data yielded a main effect of block and a Block x Congruency interaction, smaller *p* < 0.014, *F*_(15, 210)_ = 2.1, η^2^_p_ = 0.128. The congruency main effect was not significant, *F*_(1, 14)_ = 3.9, *p* < 0.066, η^2^_p_ = 0.222. The source of the Block × Congruency interaction in younger participants' data was located using simple main-effects analysis with separate error-terms for each level of block. This showed responding of Group YC to be superior to that of Group YI on blocks, 5, 6, 7, and 8, largest *p* < 0.049, *F*_(1, 14)_ = 4.6, η^2^_p_ = 0.248.

The corresponding 2 × 16 ANOVA on older participants' data yielded only a main effect of block, *F*_(15, 210)_ = 5.7, *p* < 0.001, η^2^_p_ = 0.288. Neither the congruency main effect nor its interaction with block was significant, *F*s < 1.

## Discussion

We sought to test Honey et al.’s (2010) claim that acquired equivalence of configural learning and intra-dimensional/extra-dimensional set-shifting experiments may employ a common mechanism. We reasoned that because performance at attentional set-shifting is reduced in healthy, relatively aged subjects (Owen et al., 1991; Barense et al., 2002), if Honey et al.'s assertion is correct, performance at acquired equivalence should also be reduced. Our new findings supported that suggestion. They do not unambiguously confirm that there is a relationship between configural learning and intra-dimensional/extra-dimensional set-shifting (e.g., one brought about by their reliance on a common psychological process). For example, configural learning and set shifting could be governed by independent psychological processes, each being affected by some aspect of ageing. Nonetheless, our new results represent a first and necessary step in the conclusion that configural learning and set shifting are governed by a common process.

The force of that argument relies on the specificity of the reduction in performance. That is, older participants' reduced performance at an acquired equivalence task is not theoretically decisive if it is part of a more general pattern of reduction. This could be obtained by some general disadvantage, perhaps a reduction in working memory performance, inhibition or simply less familiarity with computer-based tasks than the younger participants. Participants may have differed in their motivation to participate (younger participants gained course credit, older participants did not) or in the level or style of their educations (younger participants were current university students, older participants were not). Examination of performance that is not part of the acquired equivalence task is key to evaluating these possibilities. Older participants' performance in Experiment 2 did indicate some general deficit in discrimination relative to younger participants (which was evident before data were normalized). Of course, older people may present a general change in performance that is especially pronounced in acquired equivalence tasks. Such an interaction between tasks and the effects of age on performance could generate the results obtained. We cannot eliminate such an account but we noted above that the general performance deficiency exhibited by older participants appeared to be a response bias and rather than a discrimination deficiency. Further evidence against the suggestion that the age-associated change in acquired equivalence is merely part of a general decline comes from Experiment 1. Here, the acquired equivalence deficit was not accompanied by a general change in performance (e.g., the initial ANOVA did not generate any significant statistics that involved the variable age). We have no ready explanation for the inconsistency across experiments of the age-related response bias; but because it is uncorrelated with effects on acquired equivalence it is, without some additional elaboration, an inadequate explanation of our findings.

**Figure 6:**
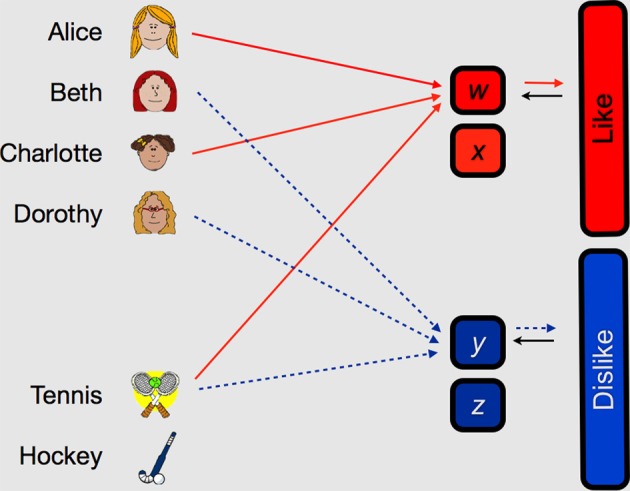
**Depiction of theoretical analysis of acquired equivalence.** The three-layered network is composed of: input units, that represent the individual components of each stimulus of each trials (e.g., both Alice and tennis); hidden units, that represent the combination of stimuli on that trial (e.g., Alice with tennis); and the output units, that represent the trial's correct outcome (e.g., that Alice likes tennis). Input unit → hidden unit and hidden unit → output unit connections begin with weights of random strength that approximate zero. Weight changes occur as learning progresses. An output unit → hidden unit connection gives feedback to the hidden unit about the trial's outcome.

Leaving to one side for a moment the age-related effects of performance, the findings of the acquired equivalence of configural learning may be accommodated by a connectionist model (Honey et al., 2010), whose main features are summarized in Figure 6. Individual elements of the discrimination, here the characters and the sports, are represented at the input layer of the network. Presentation of two items (e.g., Alice and tennis) will tend to generate activity in the network's hidden layer. Hidden-unit activity is subject to a “winner-take-all” process in which the single most active unit will suppress activity in less active units. At first, hidden unit selection will be stochastic: one lucky unit (e.g., “w”) will be active when activity is generated by the outcome (here, the information being that the character likes or dislikes the sport). The development of hidden-unit → output unit connection strength will be supported by the co-occurrence of activity sustained by “feedback” from the output unit back to the hidden unit. It is this feedback process that give this model its capacity to accommodate acquired equivalence. On a correctly answered trial (e.g., one corresponding to “Alice likes tennis”), after some training, “Alice” and “tennis” will generate activity in the hidden unit, “w,” which will generate activity in the “like” hidden unit. Here “like” is also the outcome of the trial (i.e., the participant is informed that “Alice likes tennis”). This “correct” outcome will tend to stimulate further activity, via a feedback connection, to hidden unit “w.” If we ignore any intervening trials and consider next what will happen when the participant receives a trial in which they are asked if Charlotte likes tennis. The presence of tennis in the input layer will tend to provoke activity in the hidden unit “w,” which codes for liked character-sport combinations; w's activity now provokes activity in the like output unit. On this occasion the participant is likely to correctly indicate that “Charlotte likes tennis,” which will again provoke like → “w” feedback and will improve connection strength between Charlotte and “w” and between tennis and “w.” Of course, some intervening trials will involve tennis also being disliked by some characters (viz., Beth and Dorothy). Thus, at intermediate points of training there is no reason to suppose that the presence of tennis on an “Alice likes tennis” trial will correctly activate hidden unit “w”: it could equally well activate hidden unit “y” (which codes for Beth and Dorothy's dislike of tennis). On such trials in which “y” is incorrectly selected, the dislike output-unit will be activated by “y” but the actual trial outcome will activate the like output-unit. This means that the output-unit → hidden-unit feedback signal will not sustain activity in the hidden unit “y,” and the capacity of Alice to activate it will diminish. Over multiple trials these processes will tend to encourage sharing of hidden units, thus generating acquired equivalence.

It follows from the analysis above that the disruption of the conjoint hidden- and input-unit activity on correct trials will lead to a reduction in the sharing of hidden units. Understanding such a process may be key to understanding age-related changes seen in acquired equivalence here, and, by extension, those seen in attentional set experiments (e.g., Owen et al., 1991; Barense et al., 2002). One way that this could occur has already been proposed to explain similar effects of neural manipulations on configural acquired equivalence (e.g., Coutureau et al., 2002; Iordanova et al., 2007). Here, older people's networks function as described above but with the single exception that the feedback signal from the output-layer to the hidden-layer is weakened or is absent. As outlined above, the feedback signal is a necessary step in the sole means by which input units come to share hidden units; the absence of this signal will, therefore, prevent sharing of hidden units and, therefore, prevent acquired equivalence. The absence of shared hidden units will not prevent the engagement of (trial-unique) hidden units in learning. The hidden unit that is most active on a particular trial will still tend to become associated with the output unit and it will not be activated by any other trial type. The mechanism of learning in older people, then, becomes like that described by models such as those of Rescorla (1976) and Pearce (2002): each unique combination of character and sport will require its own, unique hidden-unit. The translation from this model to ageing people is unclear but it seems possible that they reflect developmental changes in cortical regions in rhinal (e.g., Coutureau et al., 2002 or prefrontal brain-regions e.g., Iordanova et al., 2007)

Whatever the precise detail of the deficit in performance, our current results demonstrate the generality of demonstrations of acquired equivalence reported by others (e.g., Ward-Robinson and Honey, 2000; Honey and Ward-Robinson, 2001; Coutureau et al., 2002; Hodder et al., 2003; Iordanova et al., 2007) and its absence in older participants. We noted also that the parallel between these facts and age-related deficits in performance on intra-dimensional/extra-dimensional set tasks (e.g., Owen et al., 1991; Barense et al., 2002) could be the result of their being underpinned by a shared mechanism and that this does not require an attentional component (cf., Honey et al., 2010).

### Conflict of interest statement

The authors declare that the research was conducted in the absence of any commercial or financial relationships that could be construed as a potential conflict of interest.
